# Ca^2+^ affects the hyphal differentiation to sclerotia formation of *Athelia rolfsii*

**DOI:** 10.1128/spectrum.00200-24

**Published:** 2024-04-30

**Authors:** Kuan-Yu Pan, Hsien-Hao Liu, Min-Nan Tseng, Hao-Xun Chang

**Affiliations:** 1 Department of Plant Pathology and Microbiology, National Taiwan University, Taipei City, Taiwan; 2 Kaohsiung District Agricultural Research and Extension Station, Ministry of Agriculture, Pingtung, Taiwan; 3 Master Program for Plant Medicine, National Taiwan University, Taipei City, Taiwan; 4 Center for Biotechnology, National Taiwan University, Taipei City, Taiwan; University of Wisconsin-Madison, Madison, Wisconsin, USA

**Keywords:** *Athelia rolfsii*, calcium ion, *Macrophomina phaseolina*, microsclerotia, reactive oxygen species (ROS), RNA-Seq, sclerotia, *Sclerotinia sclerotiorum*, transcriptome

## Abstract

RNA-Sequencing (RNA-Seq) and transcriptomic analyses have become powerful tools to study the developmental stages of fungal structures scuh as sclerotia. While RNA-Seq experiments have been set up for many important sclerotia- and microsclerotia-forming fungi, it has not been implemented to study *Athelia rolfsii*, which is one of the earliest fungi used in literature to uncover the roles of reactive oxygen species (ROS) in stimulating sclerotia formation. This study applied RNA-Seq to profile gene expression in four developmental stages of *A. rolfsii* sclerotia. Surprisingly, gene ontology and expression patterns suggested that most ROS-scavenging genes were not up-regulated in the stages from hyphal differentiation to the initial sclerotia stage. Using antioxidant and oxidant-amended culture assay, the results suggested none of the ascorbic acid, dithiothreitol (DTT), H_2_O_2_, or superoxide dismutase inhibitors [diethyldithiocarbamate (DETC), NaN_3_, and sodium dodecyl sulfate] affected the sclerotia number. Instead, only glutathione reduced the sclerotia number. Because glutathione has also been suggested to facilitate Ca^2+^ influx, therefore, glutathione culture assays with the combination of CaCl_2_, Ca^2+^–chelator egtazic acid, DETC, and H_2_O_2_ were tested on *A. rolfsii*, as well as two other fungi (*Sclerotinia sclerotiorum* and *Macrophomina phaseolina*) for comparison. Although the addition of CaCl_2_ caused sclerotia or microsclerotia reduction for all three fungi, the CaCl_2_–ROS interaction was only observed for *S. sclerotiorum* and *M. phaseolina*, but not *A. rolfsi*. Collectively, this study not only pointed out a conserved function of Ca^2+^ in suppressing fungal sclerotia and microsclerotia formation but also highlighted sclerotia formation of *A. rolfsii* being only sensitive to Ca^2+^ and independent of ROS stimuli.

**IMPORTANCE:**

Management for plant diseases caused by soil-borne fungal pathogens is challenging because many soil-borne fungal pathogens form sclerotia for long-term survival. Advanced understanding of the molecular and cellular mechanisms of sclerotia formation may provide novel insights to prevent these fungal residues in fields. This study discovered that Ca^2+^ acts as a negative signal cue to suppress sclerotia and microsclerotia formation in three economically important fungal pathogens. Moreover, the southern blight fungus *Athelia rolfsii* appears to be only regulated by Ca^2+^ but not reactive oxygen species. Accordingly, *A. rolfsii* can be a useful system for studying the detailed mechanism of Ca^2+^, and the applicability of Ca^2+^ in reducing sclerotia could be further assessed for disease management.

## INTRODUCTION

Sclerotia are specialized structures aggregated from fungi hyphae. Most sclerotia have an interior condensed mycelia protected by a hard exterior layer to assure fungal survival in harsh environments (1); therefore, sclerotia can serve as the primary inoculum in the new agricultural season (2). There are at least 45 and 40 genera of fungi capable of producing sclerotia in the phylum Ascomycota and Basidiomycota, respectively. Among these, 25 genera are important plant pathogenic fungi, including but not limited to *Athelia*, *Colletotrichum*, *Macrophomina*, *Rhizoctonia*, *Sclerotinia*, and *Verticillium* (3). The morphology of sclerotia varies significantly among different fungal species. One classification categorizes sclerotia by size, separating them into large sclerotia, sclerotia, small sclerotia, and microsclerotia. Specifically for sclerotia, they can be further classified into the loose type, the terminal type, and the lateral type, based on the observation of hyphal intertwining and the degree of internal layering (4). The loose type is the most primitive sclerotia, formed by loosely interwoven hyphae with thicker and highly septated barrel-shaped cells. For example, *Rhizoctonia solani* AG-7 is a representative example. The terminal type sclerotia are formed by short and dichotomous branching in the hyphal tips, with the fungus *Sclerotinia sclerotiorum* as a representative example. The lateral type sclerotium is formed by one or multiple primary hyphae that create a central strand from which peripheral hyphae branch and intertwine. This type exhibits prominent internal layering, with the fungus *Athelia rolfsii* as a representative (4–6). Regardless of sclerotia types, these survival structures all serve as the dormancy and primary dispersal structures for many plant pathogenic fungi that cause economically important plant diseases, such as rice sheath blight (7), Verticillium wilt (8), charcoal rot (9), and southern blight (10). Therefore, studies on sclerotia formation may provide novel insights to prevent their formation and achieve sustainable disease management.

The morphological diversity of sclerotia not only exists among fungal species but also presents within the same species. For example, sclerotial number, sclerotial size, and sclerotial color of different *A. rolfsii* isolates have been found to vary in degree (11–13). While variation in sclerotia morphology has also been reported in *S. sclerotiorum*, limited studies were focused on the population genetics of sclerotia-forming capability. Recently, a study on the field collection of *R. solani* AG-7 isolates revealed a heritability of 0.92 and 0.31 for sclerotial number and sclerotial size, respectively (14). The results indicated that environmental factors also play important roles in sclerotial morphology. Indeed, there are several environmental stimuli, including light, temperature, nutrients, and pH, being shown to affect sclerotia formation. For instance, light regulates fungal circadian rhythms (15), and studies have suggested that light induces sclerotia production of *A. rolfsii* (16, 17). The light-sensing protein encoded by gene *veA* was essential for sclerotia formation in *Aspergillus parasiticus* and *Aspergillus flavus* (18, 19). Although some studies have suggested that light does not affect the number of sclerotia (20, 21), experimental differences such as light intensity or wavelength across studies make it hard to be conclusive (22, 23). Temperature also acts as a stimulus to affect sclerotia formation in *Villosiclava virens* (24). In addition, nutrients such as carbon sources and inorganic salts were shown to be essential for sclerotia formation (25). The C:N ratio in the culture media can affect the number and size of sclerotia (26, 27). Moreover, most studies indicated that a weakly acidic environment is suitable for sclerotia formation (28–30). For example, it has been shown that *S. sclerotiorum* releases oxalic acid to decrease environmental pH, which favors sclerotia formation (31, 32). Similarly, *A. rolfsii* also releases oxalic acid to decrease environmental pH during sclerotia formation (33). Collectively, these studies showed that sclerotia formation is influenced by both fungal genetics and environmental stimuli.

Among these stimuli, reactive oxygen species (ROS) has been intensively studied in many sclerotia-forming fungi, and most studies have agreed on the importance of ROS on sclerotia formation. The ROS-dependent theory of sclerotia formation was initially proposed due to the observation of peroxidation on the phospholipids of the cell membrane at the sclerotial periphery of *A. rolfsii* (34). Many subsequent studies confirmed the impact of the external application of ROS on *A. rolfsii*, *R. solani*, *S. sclerotiorum*, and *Macrophomina phaseolina* (35–38), therefore, Georgiou et al. (39) summarized the model suggesting that fungal mycelia under high ROS stress undergo hyphal differentiation to form sclerotia. After the proposal of the ROS model, subsequent experiments confirmed the importance of ROS, using different experiments and approaches. For example, H_2_O_2_ was reported to stimulate sclerotia production in *A. rolfsii* and *S. sclerotiorum* (35, 36), and it was also shown to lead to the early differentiation of *R. solani* AG-1 IA sclerotia (37). Besides, O_2_^−^ was shown to be the key stimulus for microsclerotia formation of *M. phaseolina* (38). Moreover, ROS not only stimulates sclerotia formation but also sclerotial maturation. Experiments with *Sclerotinia minor* demonstrated that adding diethyldithiocarbamate (DETC), a superoxide dismutase (SOD) inhibitor which blocks the generation of H_2_O_2_ from O_2_^−^, to the culture media did not affect sclerotia number, but it altered the melanization of sclerotia (40). Knocking out the Cu-Zn SOD1 gene of *S. sclerotiorum* reduced sclerotia formation (41). Similarly, in the case of microsclerotia, the transcription factor VdYap1, which is involved in intracellular ROS balance regulation, is necessary for the microsclerotia formation of *V. dahliae* (42–44).

With the advancement and generalization of high-throughput sequencing, RNA-Sequencing (RNA-Seq) has been widely applied to investigate differential gene expression during various stages of sclerotia formation, seeking additional factors that influence sclerotia formation. For example, during the transition from mycelia to the initial sclerotia stage of *R. solani* AG-1 IA, differentially expressed gene clusters related to redox processes, oxidative enzyme activity, and antioxidant activity were identified (37). Similarly, RNA-Seq on the microsclerotia formation of *M. phaseolina* showed differentially expressed genes encoding oxidative enzymes, glutathione transferase, and NADH dehydrogenase (38). For the case of microsclerotia formation in *V. dahliae*, genes related to oxidative enzymes were significantly down-regulated, and genes associated with melanin biosynthesis were highly expressed (45). Collectively, the ROS-dependent model of sclerotia formation has been widely evaluated, and RNA-Seq examination coupled with experiments using antioxidant/oxidant-amended media has become a standard approach to understanding sclerotia formation for a fungus of interest.

*A. rolfsii* is a soil-borne pathogenic fungus belonging to the phylum Basidiomycota, and it can cause southern blight diseases primarily on legumes, cucurbits, and crucifers (46). Severe incidence of southern blight may lead to yield losses of around 25% (47) and over 80% of yield losses (48, 49). *A. rolfsii* rarely produces the sexual reproductive basidiospores; instead, it mostly produces asexual sclerotia as the primary source of field infection (50). Although *A. rolfsii* is one of the early fungal species being used to study the ROS-dependent theory of sclerotia formation, RNA-Seq transcriptomics has not been implemented. Therefore, this study aims to examine the gene expression dynamics during the sclerotia formation of *A. rolfsii* and characterize the signal cues that influence the hyphal differentiation to sclerotia formation of *A. rolfsii*.

## MATERIALS AND METHODS

### Preparation for fungal materials and RNA extraction

The fungal strains of *A. rolfsii* 1-4-03, *M. phaseolina* a31, and *S. sclerotiorum* 1980 were routinely maintained on potato dextrose agar (PDA) in the dark, with the former two at 28°C and the latter one at 25°C. To prepare fungal samples for RNA extraction, there were four stages of sclerotia formation for *A. rolfsii* defined in this study, including the mycelia stage (S0), the initial stage (S1), the developmental stage (S2), and the mature stage (S3). The S0 stage was obtained by liquid potato dextrose broth culture for 1.5 days at 28°C. For the S1, S2, and S3 samples, sclerotia collection was performed at 3 days, 4.5 days, and 6 days on PDA, respectively. The collected samples were placed in a 2-mL centrifuge tube with 1 mL of sterile water and 100 µL of 0.5-mm glass beads. They were gently vortexed using a homogenizer to remove any superficial mycelia attached on sclerotia. The S1 samples were vortexed with glass beads at 5.0 m/s. The S2 and S3 samples were vortexed with 100 µL of 1-mm zirconium beads that were used at 6.0 m/s. The supernatant containing floating mycelia was removed, and approximately 200–300 mg of purified mycelia (S0) and sclerotia (S1–S3) were ground with a mortar and pestle in liquid nitrogen with 1.5 mL of TRIzol (Invitrogen, Thermo Fisher Scientific, Waltham, MA, USA). Chloroform and isopropanol were used to purify RNA, and half volume of 8-M LiCl (Sigma-Aldrich, Burlington, Canada) was added to one volume of the RNA samples to further precipitate and purify RNA. The RNA was dissolved in the diethyl pyrocarbonate (DEPC)-treated H_2_O and measured by a NanoDrop One (Thermo Fisher Scientific) to ensure quality (A260/A280: 1.8–2.2; A260/A230 ≥ 2.0).

### RNA-Seq and transcriptome analyses

Three biological replicates of each four stages (S0, S1, S2, and S3) were included, and the RNA quality was confirmed by the Q-Sep100 instrument (Bioptic, New Taipei City, Taiwan) before sequencing with paired-end 150 bp on the Illumina NovaSeq 6000 platform (BIOTOOLS, New Taipei City, Taiwan). The sequencing results were quality-checked by FastQC version 0.11.9, and the adapter sequences and low-quality sequences (Phred score <30) were filtered by Cutadapt version 2.3 (51). The reference genome of *A. rolfsii* Mr10 strain available in the NCBI database (52) was used for *ab initio* gene prediction using AUGUSTUS version 3.4.0 with *Ustilago maydis* being used as the model organism (53). Kallisto version 0.46.1 (54) and the R package Sleuth version 0.30 (55) were applied for differential expression analysis. Gene ontology (GO) terms were annotated by the OMA orthology database (56). GO enrichment analysis was analyzed using the R package clusterProfiler version 4.4.4 (57), and the results were visualized by REVIGO (58). If additional gene annotation is needed, BLAST version 2.9.0 (59) was used to BLAST against *R. solani* sequences in the NCBI nr database (February 2023).

### Quantification of sclerotial number by image analysis

The sclerotial number on each Petri dish plate was quantified after 2 weeks of culture, and the plates were photographed by the VideGel image analysis system (TOPBIO, New Taipei City, Taiwan). The parameter settings were exposure time = 0.11 s and zoom lens = 15 mm. Subsequently, the software ImageJ version 1.53 was used to calculate the number of sclerotia. Firstly, the image was gray-scaled with an 8-bit color scale, the parameter threshold = 120, and the built-in function “Analyze Particles” of ImageJ. Subsequently, the plate area was selected manually at the default settings for pixel size and circularity. Finally, the “Watershed” function was applied to divide the connected dots and then quantified the number of dots as sclerotia. In the case of DTT treatment, quantification was conducted manually due to heavy pigmentation resulting from the chemical treatments.

### The ROS assays and staining

For ROS inhibition assay, three antioxidants including ascorbic acid (Panreac, Barcelona, Spain), DTT (BioShop, Burlington, Canada), and glutathione (BioShop) were tested. The antioxidant stock solutions were prepared and sterilized by filtering through 0.22-µm syringe filters and then amended into sterilized PDA to acquire the desired concentration for experiments. For ROS stimulation assay, 30% H_2_O_2_ (Panreac), sodium azide (NaN_3_) (BioShop), sodium DETC (Alfa Aesar, Tewksbury, MA, USA), and sodium dodecyl sulfate (SDS) (BioShop) were tested. The oxidant solutions were adjusted to the desired concentration before being amended into sterilized PDA. Alternatively, the oxidant solutions were immersed on top of the 2.5-day-old mycelia in PDA plates for 10 minutes. The solutions were drained, and the culture plates were maintained in the dark at 28°C for sclerotia formation. For each antioxidant or oxidant, there were five biological replicates (plates) in each experiment, and the experiments were repeated three times. Meanwhile, the mycelial growth rate was measured at a 12-hour interval to evaluate chemical effects on fungal growth. The optimal concentration for each antioxidant or oxidant was determined by the maximum concentration that can be applied without significantly reducing mycelial diameter on PDA plates.

ROS accumulation was visualized by staining hyphae or sclerotia with nitroblue tetrazolium (NBT) (BioShop) and diaminobenzidine (3′,3′-diaminobenzidine, DAB) (AK Scientific, Ahern Ave, CA, USA). The 0.05% (wt/vol) NBT was dissolved in the 70% dimethyl sulfoxide. The 0.05% (wt/vol) DAB was prepared in H_2_O with slow addition of HCl to facilitate dissolution (40). An equal volume of phosphate-buffered saline was added to NBT and DAB solutions, before filtering through 0.22-µm syringe filters. The final pH of NBT and DAB dyes were 8 and 7.5, respectively.

### The calcium assay and CaCl_2_–ROS interaction assay

For the calcium assay, CaCl_2_ (Panreac) was used as the exogenous Ca^2+^ source, and egtazic acid (EGTA) (BioShop) was used as the Ca^2+^ chelator. The EGTA stock solution was prepared in H_2_O with adjusted pH to 8, and the EGTA was added into sterilized PDA to obtain a final concentration of 0.5 mM at pH 7. The mycelial diameter was also measured at a 12-hour interval, and the optimal concentration for CaCl_2_ was determined by the maximum concentration that can be applied without significantly reducing mycelial diameter. For the calcium assay, there were five biological replicates (plates) in each experiment, and the experiments were repeated three times.

For CaCl_2_–ROS interaction assay, the CaCl_2_ added in PDA for *A. rolfsii*, *M. phaseolina*, and *S. sclerotiorum* was 30 mM, 40 mM, and 10 mM, respectively. Image analysis was adapted to quantify the number of microsclerotia of *M. phaseolina* (38). In brief, the mycelial plugs were sampled from 3.5 cm from the center when the 9-cm PDA plate was fully covered by mycelia. The image under a dissecting microscope was photographed by the White IMX-2000 imaging system (WHITE LTD., Taipei, Taiwan), and ImageJ was used to quantify the abundance of microsclerotia with a setting of 8-bit grayscale and a parameter threshold = 100, using the built-in “Analyze Particles” function at the default settings for pixel size and circularity. In addition, the entire Petri dish plate was also photographed to observe the final abundance of microsclerotia after 7 days. As for *S. sclerotiorum*, the number of sclerotia was counted manually after 7 days. For the CaCl_2_–ROS assay, there were five biological replicates (plates) in each experiment, and the experiments were repeated three times.

### Statistics

Statistical analyses were performed in R environment 4.2.0 loading the packages “agricolae,” “car,” and “FSA.” The original measurement data were tested for normality using the Shapiro–Wilk test and for the homogeneity using the Bartlett’s test or Levene’s test. If the data fulfill the parametric assumptions, analysis of variance and Tukey’s honestly significant difference tests were applied. Alternatively, non-parametric Kruskal–Wallis test and Dunn’s test were applied. The significance was determined by the Benjamini-Hochberg-adjusted *P* value at 0.05.

## RESULTS

### RNA-Seq and GO enrichment analyses on the four stages of sclerotia formation

**Fig 1:**
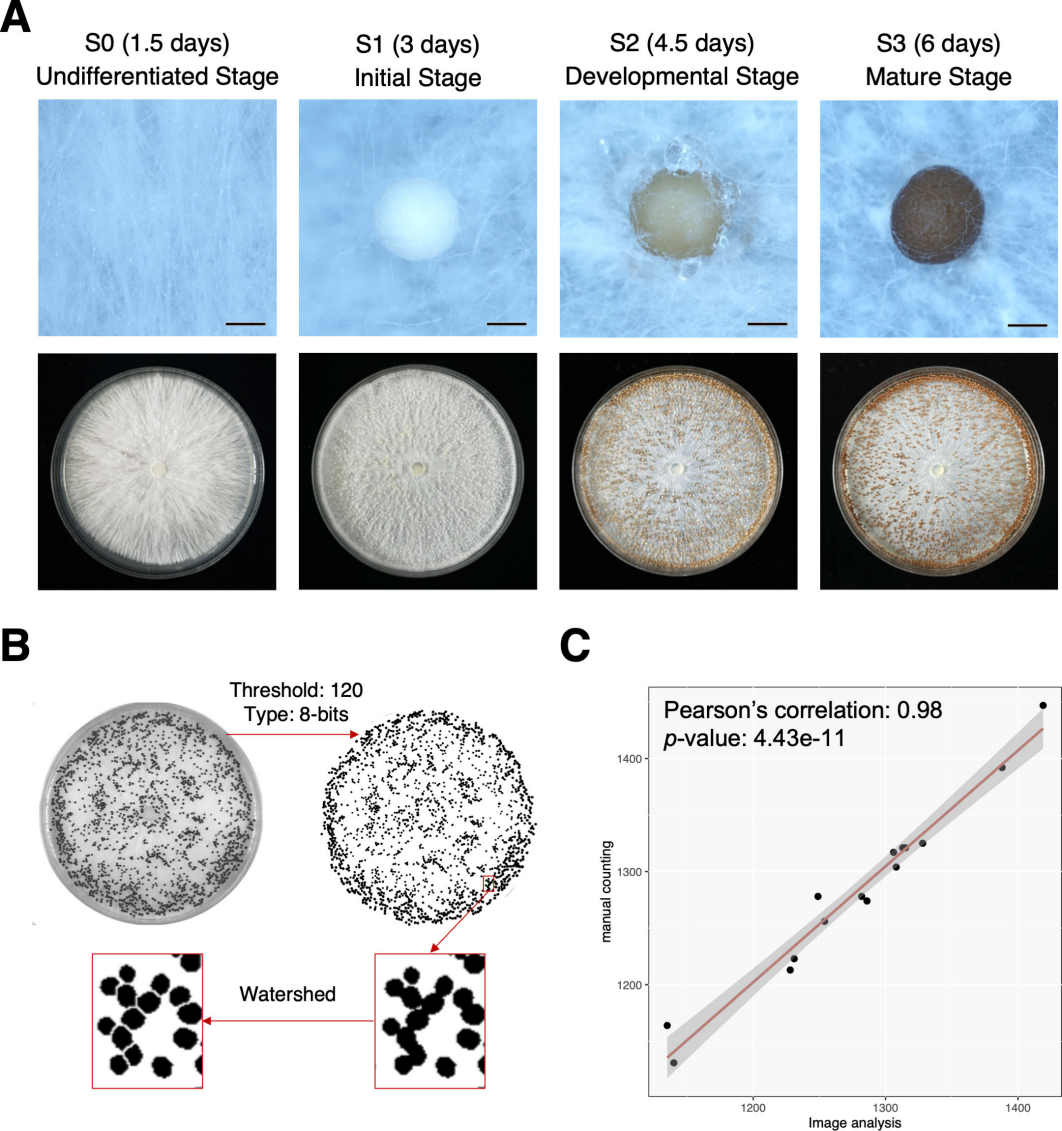
Morphological assessment and quantification of sclerotia formed by *A. rolfsii*. (**A**) Four stages of sclerotia formation were determined. The S0 stage represents the undifferentiated mycelia. The S1 stage represents the aggregated mycelial balls without pigmentation. The S2 stage represents the developing sclerotia with light pigmentation and exudates on the surface. The S3 stage represents the mature sclerotia, which are dark pigmented and dehydrated. (**B**) The image-based quantification with settings to distinguish individual sclerotia of *A. rolfsii*. (**C**) The results of image-based quantification and naked eye counting resulted in a high and significant correlation.

The mycelia of *A. rolfsii* at 3 days post-subculture on PDA plates began to aggregate and form a fluffy mycelial ball, which is defined as the initial stage of sclerotia (S1 stage). These white and loose mycelial balls started to form an exterior rind with light brown pigmentation at 4.5 days. Meanwhile, there were some fungal exudates accumulated on the surface of sclerotia at this developmental stage (S2 stage). These sclerotia eventually matured at 6 days, and the mature stage of sclerotia (S3 stage) exhibited dehydrated and solidified rind with dark pigmentation (Fig. 1A). Although sclerotia of *A. rolfsii* are large enough for manual counting, an image analysis condition was developed for quantifying the abundance of sclerotia efficiently (Fig. 1B). Based on the optimization of image settings, the Pearson’s correlation between image-based results and manual counting was 0.98 (Fig. 1C), which indicated the robustness of image analysis on quantifying sclerotia number.

**Fig 2:**
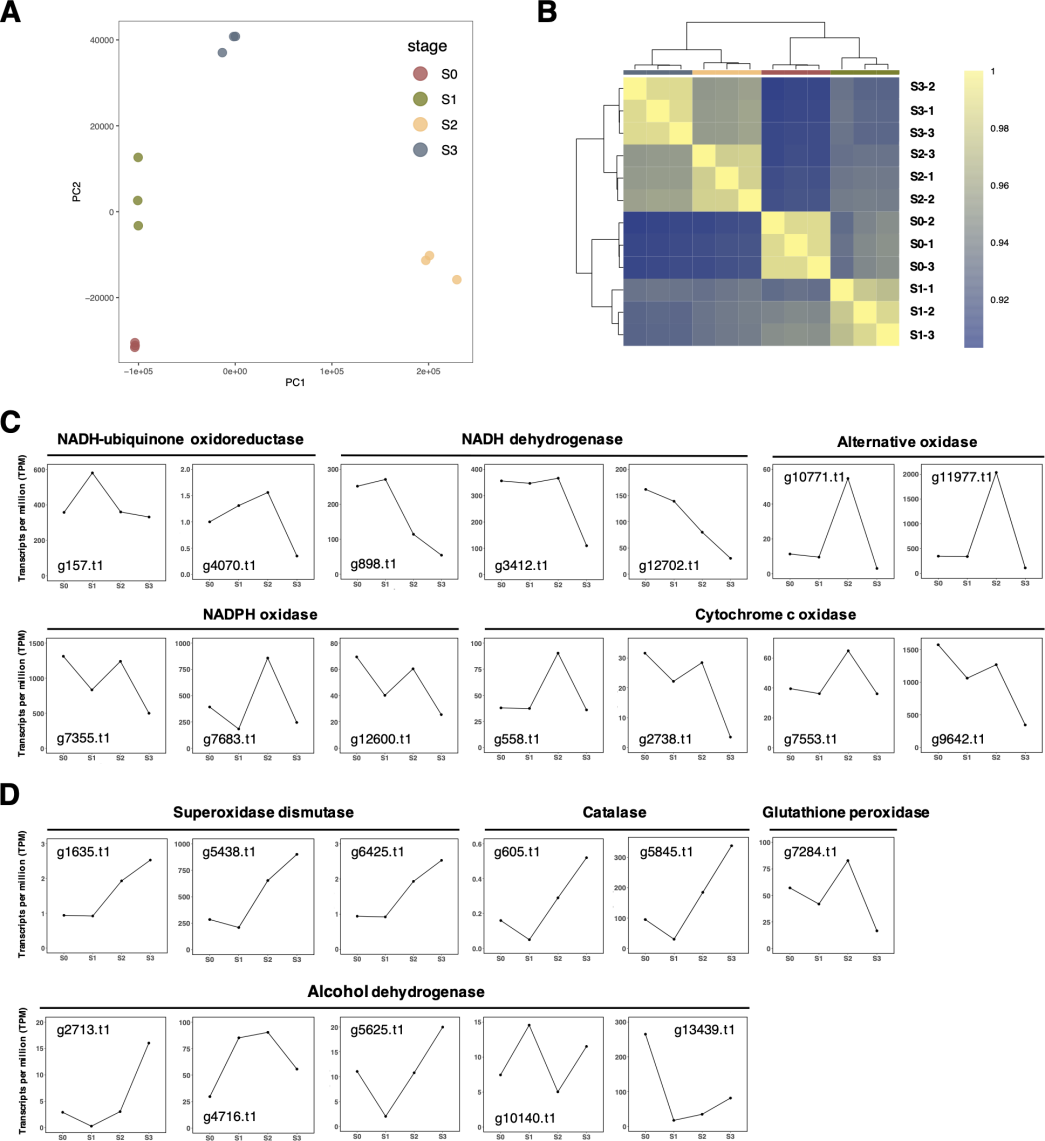
Transcriptomic analyses and the ROS-related gene expression at the four stages of sclerotia formation. (**A**) The principal component analysis shows a clear grouping of the four stages, indicating that the robustness of experimental setup and the expression variation may be mainly explained by the developmental stages. (**B**) The Jensen–Shannon divergence analysis shows distinct categories among the four stages. The expression patterns at the S2 and S3 stages were more similar to each other, differing from the S0 or S1 stages. (**C**) The expression patterns of genes annotated with ROS-generating enzymes. (**D**) The expression patterns of genes annotated with ROS-scavenging enzymes.

The RNA-Seq experiment resulted in 42–57 million reads per sample qualified for analyses (data not shown). The principal component analysis exhibited a clear sample separation among the S0 to S3 stages (Fig. 2A). The Jensen–Shannon divergence analysis suggested that the S0 and S1 samples had a closer expression pattern, while the S2 and S3 samples had a closer expression pattern (Fig. 2B). Collectively, the RNA-Seq samples together presented a distinct grouping to capture meaningful differential expressed genes between stages and study *A. rolfsii* genes involved in sclerotia formation.

Focusing on the S1 versus S0 stage, 2,633 and 2,675 genes were being up-regulated and down-regulated, respectively. Two GO terms (GO:0016614 and GO:0016616) related to the oxidoreductase activity were found to be enriched in the transition stage between S0 and S1 (data not shown). For the S2 versus S1 stage, 2,464 and 2,591 genes were being up-regulated and down-regulated, respectively. One GO term (GO:0016209) related to the antioxidant activity and two GO terms (GO:0016860 and GO: 0016684) related to the oxidoreductase activity were found to be enriched in the transition stage between S1 and S2 (data not shown). Lastly, for the S3 versus S2 stage, 3,143 and 3,185 genes were being up-regulated and down-regulated, respectively. One GO term (GO:0015453) related to the oxidoreduction-driven active transmembrane transporter activity and seven GO terms (GO:0016651, GO:0016667, GO:0016860, GO:0016655, GO:0016653, GO:0016717, and GO:0016864) related to the oxidoreductase activity were found to be enriched in the transition stage between S2 and S3 (data not shown). These results indicated that gene functions related to ROS metabolism were expressed throughout the four stages, and more ROS-related GO terms were enriched toward the latter stages of sclerotia formation.

### The ROS-related gene expression at the four stages of sclerotia formation

Targeting the ROS-generating genes in the mitochondrial electron transport chain, there were two genes annotated as NADH–ubiquinone oxidoreductases (g157.t1 and g4070.t1), three genes annotated as NADH dehydrogenase (g898.t1, g3412.t1, and g12702.t1), three genes annotated as NADPH oxidases (g7355.t1, g7683.t1, and g12600.t1), four genes annotated as cytochrome c oxidases (g558.t1, g2738.t1, g7553.t1, and g9642.t1), and two genes annotated as alternative oxidases (g10771.t1 and g11977.t1). Among these genes, only g157.t1, g4070.t1, and g898.t1 showed up-regulations from the S0 to S1 stage, and the rest of the genes showed steady or decreased expressions from the S0 to S1 stage (Fig. 2C).

Targeting the ROS-scavenging genes, there were three genes annotated as SOD (g1635.t1, g5438.t1, and g6425.t1), two genes annotated as catalase (g605.t1 and g5845.t1), one gene annotated as glutathione peroxidase (g7248.t1), and five genes annotated as alcohol dehydrogenases (g2713.t1, g4716.t1, g5625.t1, g10140.t1, and g13439.t1). These genes were all displaying steady or decreased expression from the S0 to S1 stage, except for two alcohol dehydrogenases (g4716.t1 and g10140.t1) (Fig. 2D).

In summary, for genes involved in ROS generation (Fig. 2C), most of these genes have a steady or decreased expression pattern from the S0 to S1 stage, an increased expression pattern from the S1 to S2 stage, and a decreased expression pattern from the S2 to S3 stage. On the other hand, for genes involved in the ROS scavenging (Fig. 2D), most of these genes have a steady or decreased expression pattern from the S0 to S1 stage and an increased expression pattern from the S1 to S3 stage. Collectively, the transcriptomic analyses suggested that ROS-related gene expressions and functions may be linked to the developmental and mature stages (S2 and S3) in sclerotia formation of *A. rolfsii*.

### The ROS assays and staining

**Fig 3:**
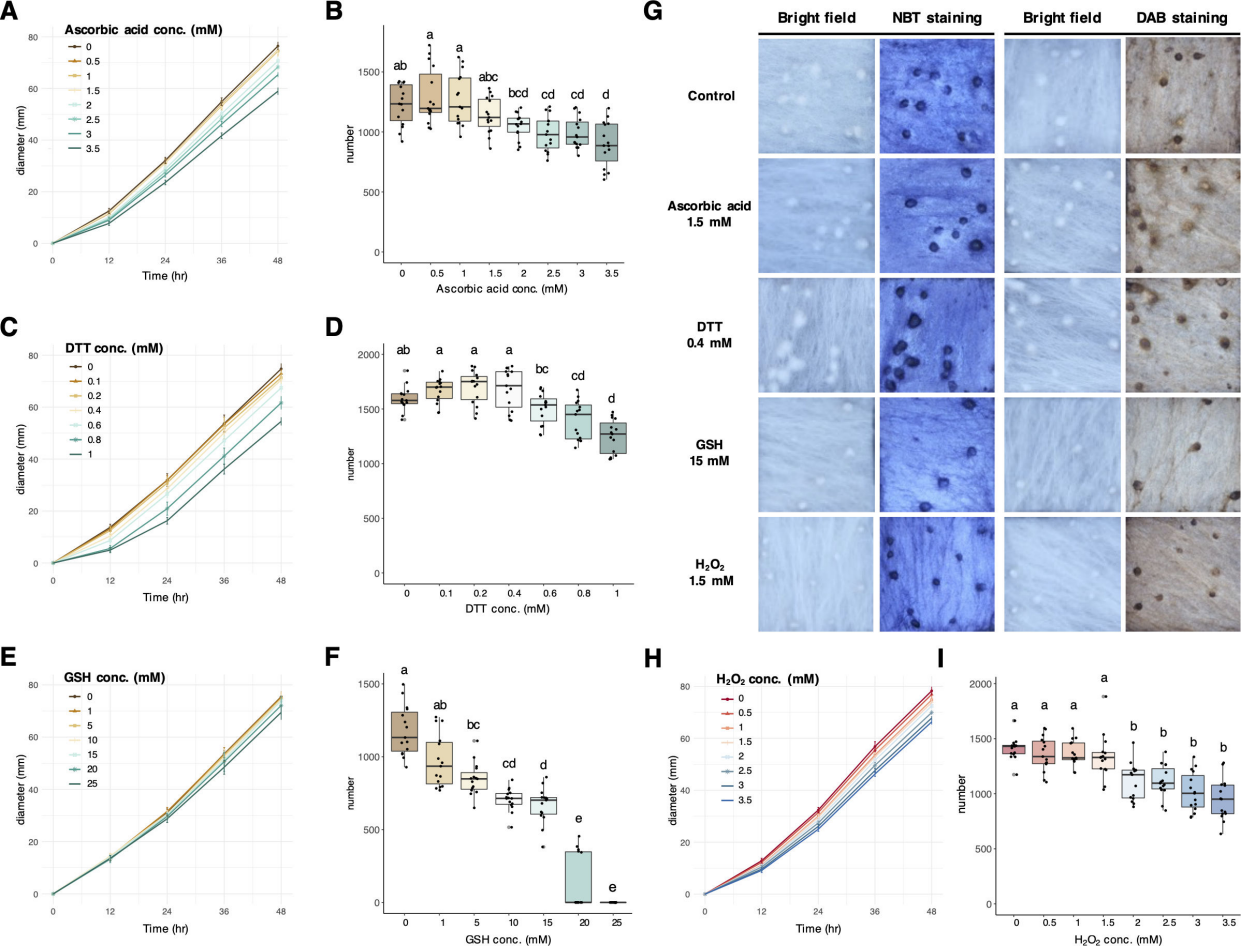
The ROS assays and staining for ascorbic acid, DTT, glutathione (GSH) and H_2_O_2_. (**A**) Serial concentrations of ascorbic acid and 1.5 mM were determined to be the optimal condition without reducing the mycelial diameter. (**B**) Serial concentrations of ascorbic acid and 1.5 mM did not reduce the sclerotial number. (**C**) Serial concentrations of DTT and 0.4 mM were determined to be the optimal condition without reducing the mycelial diameter. (**D**) Serial concentrations of DTT and 0.4 mM did not reduce the sclerotial number. (**E**) Serial concentrations of GSH and 15 mM were determined to be the optimal condition without reducing the mycelial diameter. (**F**) Serial concentrations of GSH and 15 mM reduced the sclerotial number. (**G**) NBT and DAB staining for O_2_^−^ and H_2_O_2_, respectively. While DTT and GSH may reduce the accumulation of H_2_O_2_ in mycelia, no reduction of the sclerotial number was observed in these conditions. (**H**) Serial concentrations of H_2_O_2_ and 1.5 mM were determined to be the optimal condition without reducing the mycelial diameter. (**I**) Serial concentrations of H_2_O_2_ and 1.5 mM did not increase the sclerotial number.

In order to test the involvement of ROS in sclerotia formation, three antioxidants including ascorbic acid, DTT, and glutathione (GSH) were selected in the ROS inhibition assay. After searching for the optimal concentration, the maximum concentration without inhibiting mycelial growth was determined at 1.5 mM, 0.4 mM, and 15 mM for ascorbic acid, DTT, and GSH, respectively (Fig. 3A, C, and E). At these concentrations, the addition of ascorbic acid and DTT in the PDA plates caused no difference regarding sclerotial number (Fig. 3B and D). However, the addition of GSH drastically reduced sclerotia number in a dosage-dependent manner (Fig. 3F). There was no observational difference in the NBT staining, indicating that the abundance of O_2_^−^ in mycelial may not be affected in the presence of these antioxidants. On the other hand, DAB staining showed lighter mycelia in the DTT and GSH treatments (Fig. 3G). Subsequently, H_2_O_2_ was amended in PDA plates at a serial concentration. However, at the maximum H_2_O_2_ concentration at 1.5 mM without inhibiting mycelial growth (Fig. 3H), the sclerotia number was not increased (Fig. 3I). Therefore, these results did not support the role of H_2_O_2_ in stimulating sclerotia formation of *A. rolfsii*.

**Fig 4:**
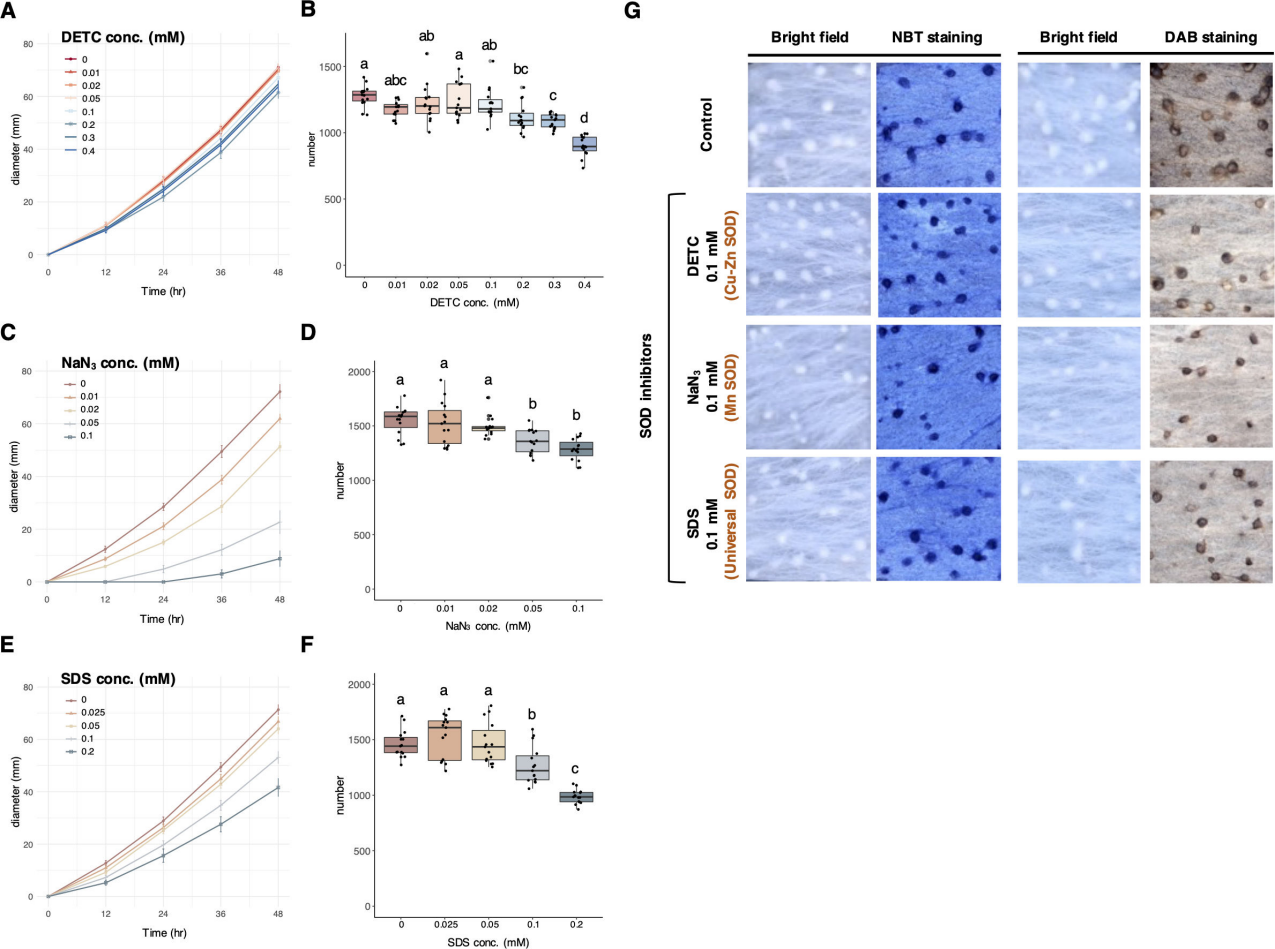
The ROS assays and staining for three SOD inhibitors, including DETC, NaN_3_, and SDS. (**A**) Serial concentrations of DETC and 0.1 mM were determined to be the optimal condition without reducing the mycelial diameter. (**B**) Serial concentrations of DETC and 0.1 mM did not increase the sclerotial number. (**C**) Serial concentrations of NaN_3_ all suppressed the mycelial diameter. (**D**) NaN_3_ above 0.05 mM did not increase the sclerotial number. (**E**) Serial concentrations of SDS all suppressed the mycelial diameter. (**F**) SDS above 0.1 mM did not increase the sclerotial number. (**G**) The SOD inhibitors (DETC, NaN_3_, and SDS) may block the transition from O_2_^−^ to H_2_O_2_, resulting in less H_2_O_2_. Nonetheless, the accumulation of O_2_^−^ did not increase the sclerotial number, indicating that O_2_^−^ may not be the signal cue for stimulating hyphal differentiation to sclerotia formation of *A. rolfsii*.

Meanwhile, three chemicals including the DETC, NaN_3_, and SDS that have been used as Cu–Zn SOD inhibitor, Mn SOD inhibitor, and universal SOD inhibitor, respectively (40, 60, 61), were selected in the ROS stimulation assay. While the maximum concentration without inhibiting mycelial growth was determined at 0.1 mM for DETC, the addition of NaN_3_ or SDS inhibited mycelial growth rate in a dosage-dependent manner (Fig. 4A, C, and E). Nonetheless, none of these treatments increased sclerotial number (Fig. 4B, D, and F). Because addition of these SOD inhibitors caused less H_2_O_2_ accumulation in the mycelia according to the DAB staining (Fig. 4G), but there was no difference in sclerotial number, the assays provided no support for O_2_^−^ as the stimulus to initiate hyphal differentiation to sclerotia formation of *A. rolfsii*.

### The calcium assay and CaCl_2_–ROS interaction assay

Because two antioxidants (ascorbic acid and DTT) and four oxidants (H_2_O_2_, DETC, NaN_3_, and SDS) did not provide evidence for O_2_^−^ or H_2_O_2_ stimulating sclerotia formation, we re-considered additional effects of GSH (62). Previous studies have reported that exogenous application of GSH can stimulate Ca^2+^ to release into cytosol (63, 64). More recently, GSH and its derivatives have been shown to dock into the extracellular binding pocket of Ca^2+^-sensing receptors (65). Therefore, we hypothesized that the GSH-amended PDA plates may cause extracellular Ca^2+^ to move into fungal hyphae and result in the reduction of sclerotial number.

**Fig 5:**
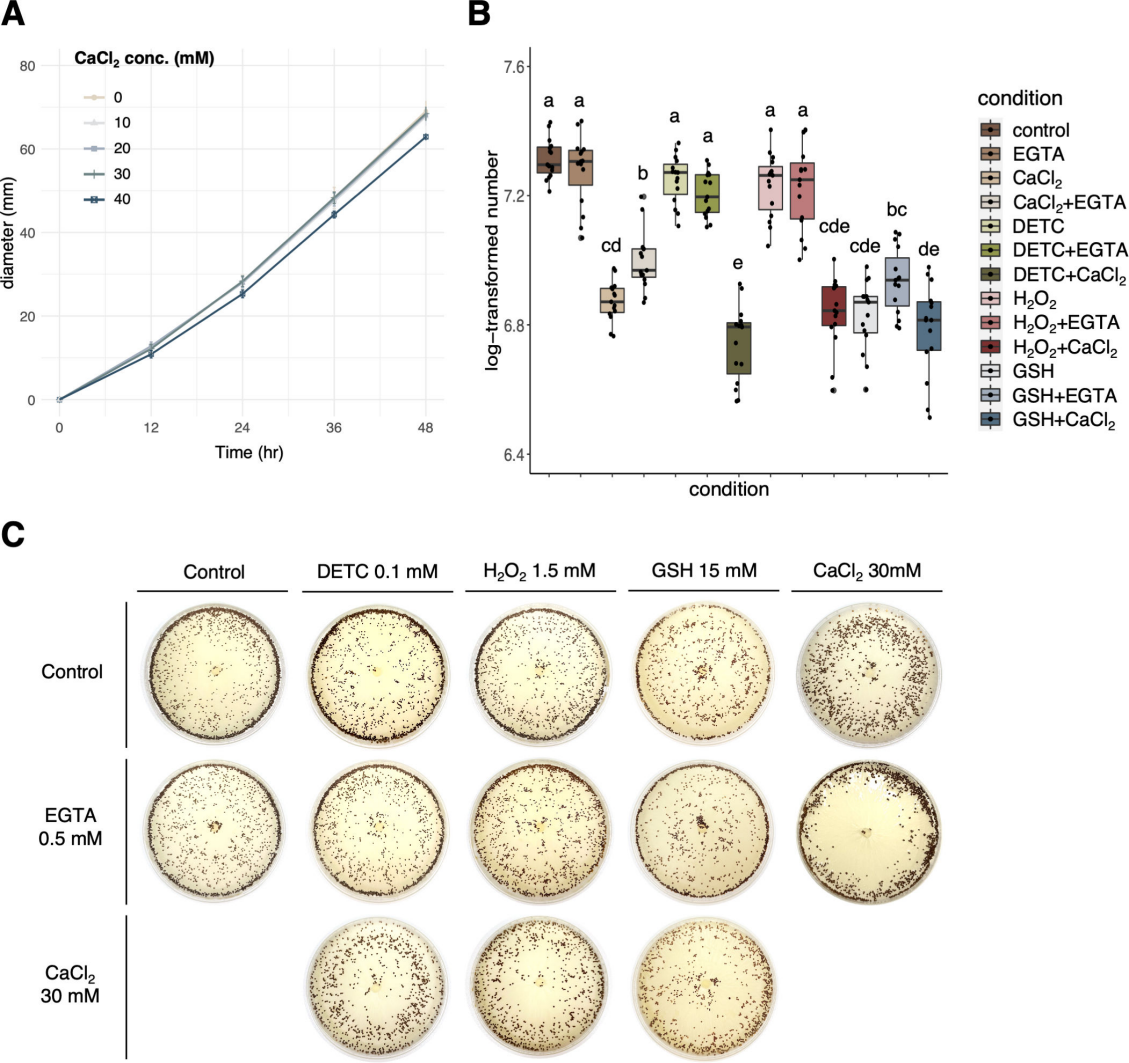
The calcium assay and CaCl_2_–ROS interaction assay for *A. rolfsii*. (**A**) Serial concentrations of CaCl_2_ and 30 mM were determined to be the optimal condition without reducing the mycelial diameter. (**B**) Quantification and statistical analysis for the individual and pairwise effects of CaCl_2_, Ca^2+^–chelator EGTA, DETC, H_2_O_2_, and GSH on the sclerotial number of *A. rolfsii*. (**C**) Colony morphology for the individual and pairwise effects of CaCl_2_, Ca^2+^–chelator EGTA, DETC, H_2_O_2_, and GSH on the sclerotial number of *A. rolfsii*. In brief, CaCl_2_ alone suppressed the sclerotial number, and the addition of EGTA partially restored the suppressing effect of CaCl_2_. The addition of DETC (resulting in O_2_^−^ accumulation) or H_2_O_2_ did not increase or decrease the sclerotial number, indicating no CaCl_2_–ROS interaction for the hyphal differentiation into microsclerotia formation of *A. rolfsii*.

To test the hypothesis, a calcium assay was set up to identify the maximum concentration of 30-mM CaCl_2_ that does not inhibit the mycelial diameter of *A. rolfsii* (Fig. 5A). Surprisingly, the sclerotial number was found to be significantly reduced (Fig. 5B). In order to double confirm the phenomenon, a Ca^2+^–chelator EGTA was amended in the PDA plates along and together with CaCl_2_. The results showed that EGTA treatment did not alter sclerotial number, but the CaCl_2_–egtazic acid (ETGA) treatment partially restored the suppression effect of CaCl_2_ (Fig. 5C). Collectively, the results demonstrated that exogenous CaCl_2_ or GSH may facilitate a Ca^2+^ influx to hyphae and suppress the hyphal differentiation to sclerotia formation.

Since studies have shown that Ca^2+^ signaling pathway cross-talks with ROS signaling pathway in various aspects, a CaCl_2_–ROS interaction assay was established to understand the potential hierarchy between these two signals. In the CaCl_2_–DETC treatment or the CaCl_2_–H_2_O_2_ treatment (Fig. 5B and C), the results showed no interference of DETC or H_2_O_2_ on the suppression effect of CaCl_2_ on the sclerotial number. Moreover, the CaCl_2_–GSH treatment did not show an additive effect, indicating that CaCl_2_ and GSH may work on the same hierarchy to suppress sclerotia formation (Fig. 5B and C).

**Fig 6:**
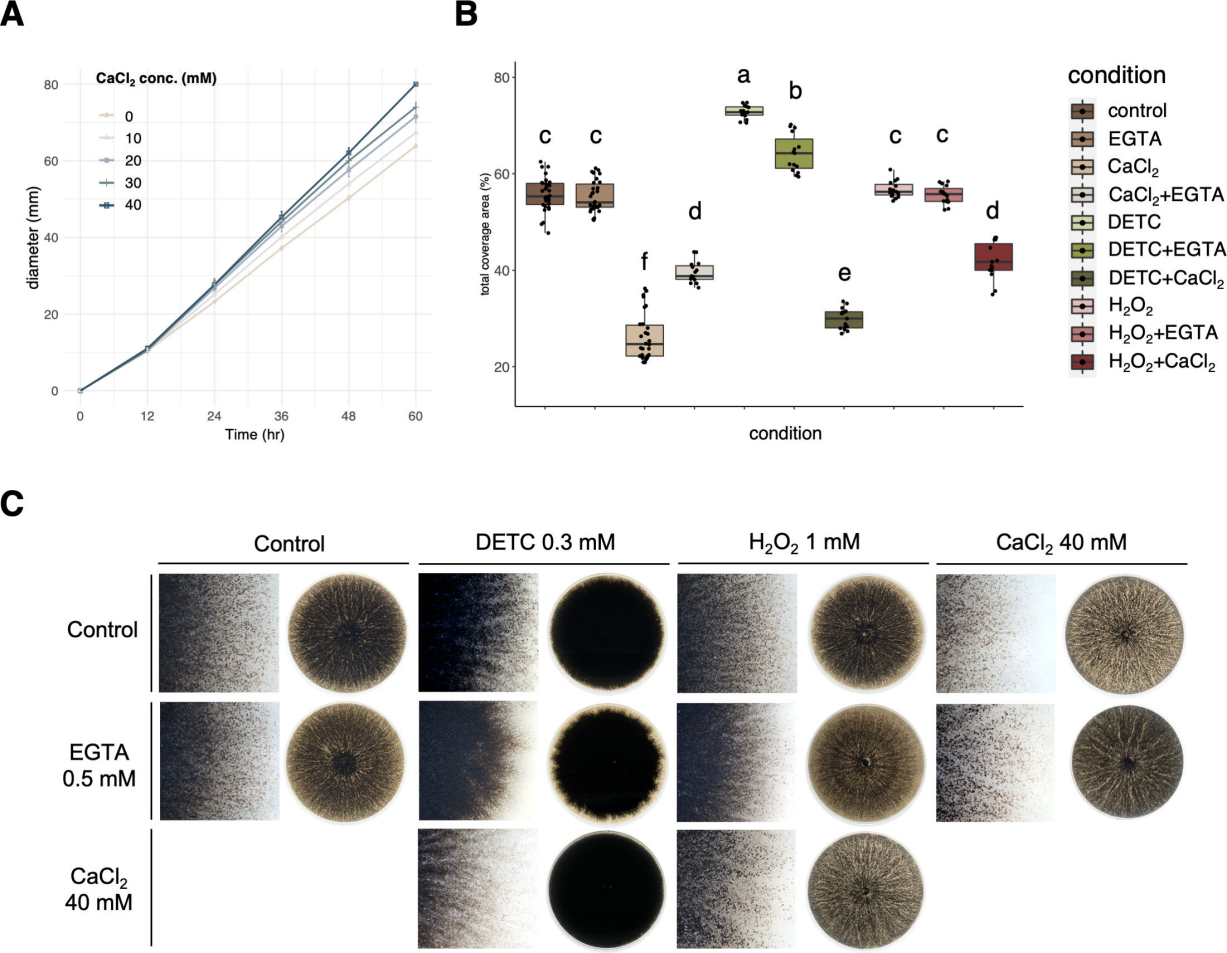
The calcium assay and CaCl_2_–ROS interaction assay for *M. phaseolina*. (**A**) Serial concentrations of CaCl_2_ and 40 mM were determined to be the optimal condition without reducing the mycelial diameter. (**B**) Quantification and statistical analysis for the individual and pairwise effects of CaCl_2_, Ca^2+^–chelator EGTA, DETC, and H_2_O_2_ on the microsclerotial number of *M. phaseolina*. (**C**) Colony morphology for the individual and pairwise effects of CaCl_2_, Ca^2+^–chelator EGTA, DETC, and H_2_O_2_ on the microsclerotial number of *M. phaseolina*. In brief, CaCl_2_ alone suppressed the microsclerotial number, and the addition of EGTA partially restored the suppressing effect of CaCl_2_. The addition of DETC (resulting in O_2_^−^ accumulation) increased the microsclerotial number. The addition of H_2_O_2_ did not increase or decrease the microsclerotial number. In the presence of DETC or H_2_O_2_, the suppressing effect of CaCl_2_ was rescued, indicating the presence of the CaCl_2_–ROS interaction for the hyphal differentiation into microsclerotia formation of *M. phaseolina*.

**Fig 7:**
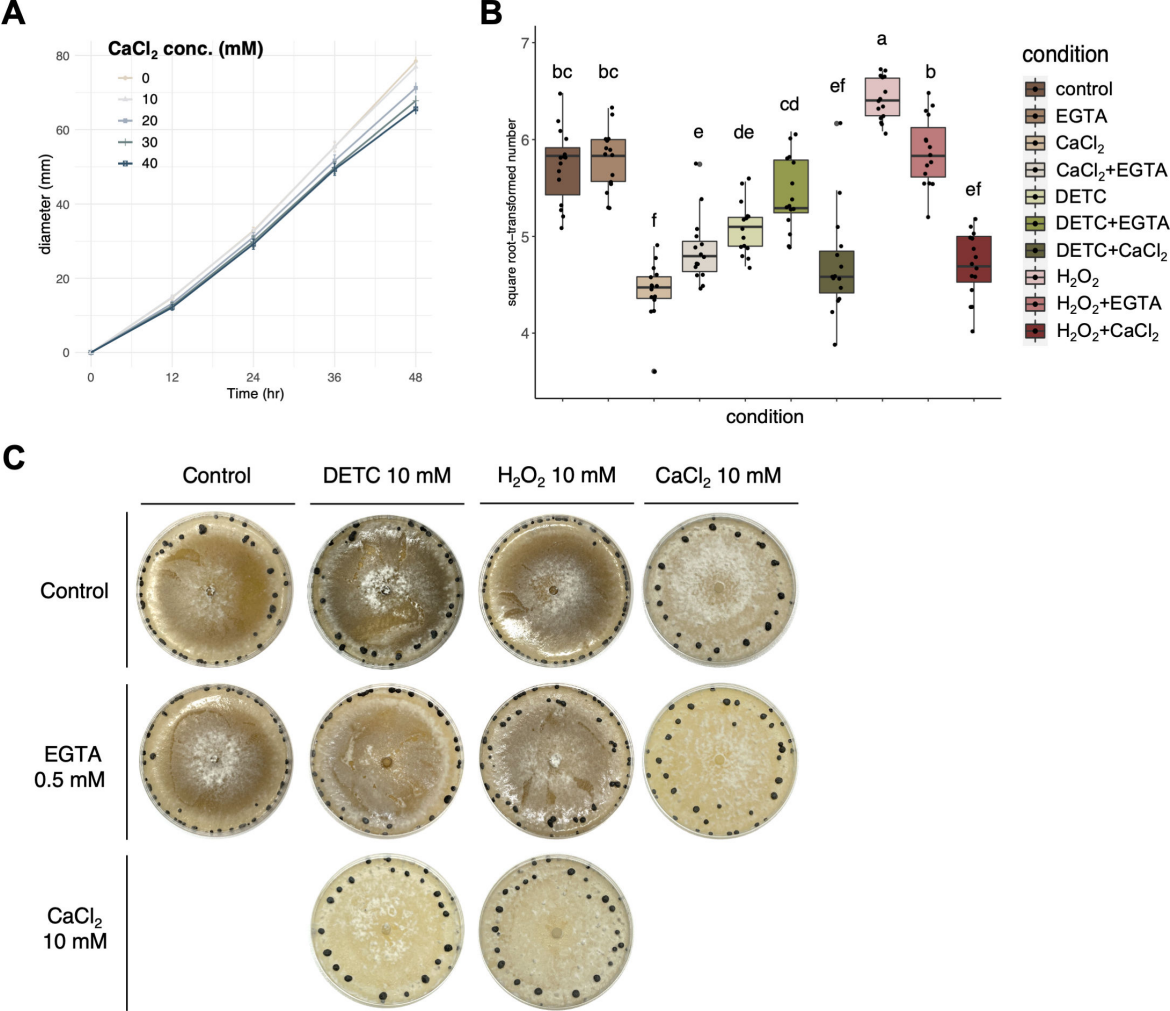
The calcium assay and CaCl_2_–ROS interaction assay for *S. sclerotiorum*. (**A**) Serial concentrations of CaCl_2_ and 10 mM were determined to be the optimal condition without reducing the mycelial diameter. (**B**) Quantification and statistical analysis for the individual and pairwise effects of CaCl_2_, Ca^2+^–chelator EGTA, GSH, DETC, and H_2_O_2_ on the sclerotial number of *S. sclerotiorum*. (**C**) Colony morphology for the individual and pairwise effects of CaCl_2_, Ca^2+^–chelator EGTA, GSH, DETC, and H_2_O_2_ on the sclerotial number of *S. sclerotiorum*. In brief, CaCl_2_ alone suppressed the sclerotial number, and the addition of EGTA partially restored the suppressing effect of CaCl_2_. The addition of DETC (blocking H_2_O_2_ generation) decreased the sclerotia number, and the addition of H_2_O_2_ increased the sclerotia number. In the presence of DETC or H_2_O_2_, the suppressing effect of CaCl_2_ was not rescued, indicating that the CaCl_2_ effect was dominant over the H_2_O_2_ effect for the hyphal differentiation into sclerotia formation of *S. sclerotiorum*.

To validate the technical appropriateness of the CaCl_2_–ROS interaction assay, two additional fungi, including *M. phaseolina* which was previously reported to be stimulated by O_2_^−^, and *S. sclerotiorum*, which was previously found to be stimulated by H_2_O_2_, were included in the experiment. The results consistently confirmed that exogeneous DETC indeed increased the microsclerotia number of *M. phaseolina* (Fig. 6A), and exogeneous H_2_O_2_ indeed increased the sclerotia number of *S. sclerotiorum* (Fig. 7A). More importantly, addition of CaCl_2_ decreased sclerotia number of both fungi, similar to *A. rolfsii* (Fig. 6B and 7B).

**Fig 8:**
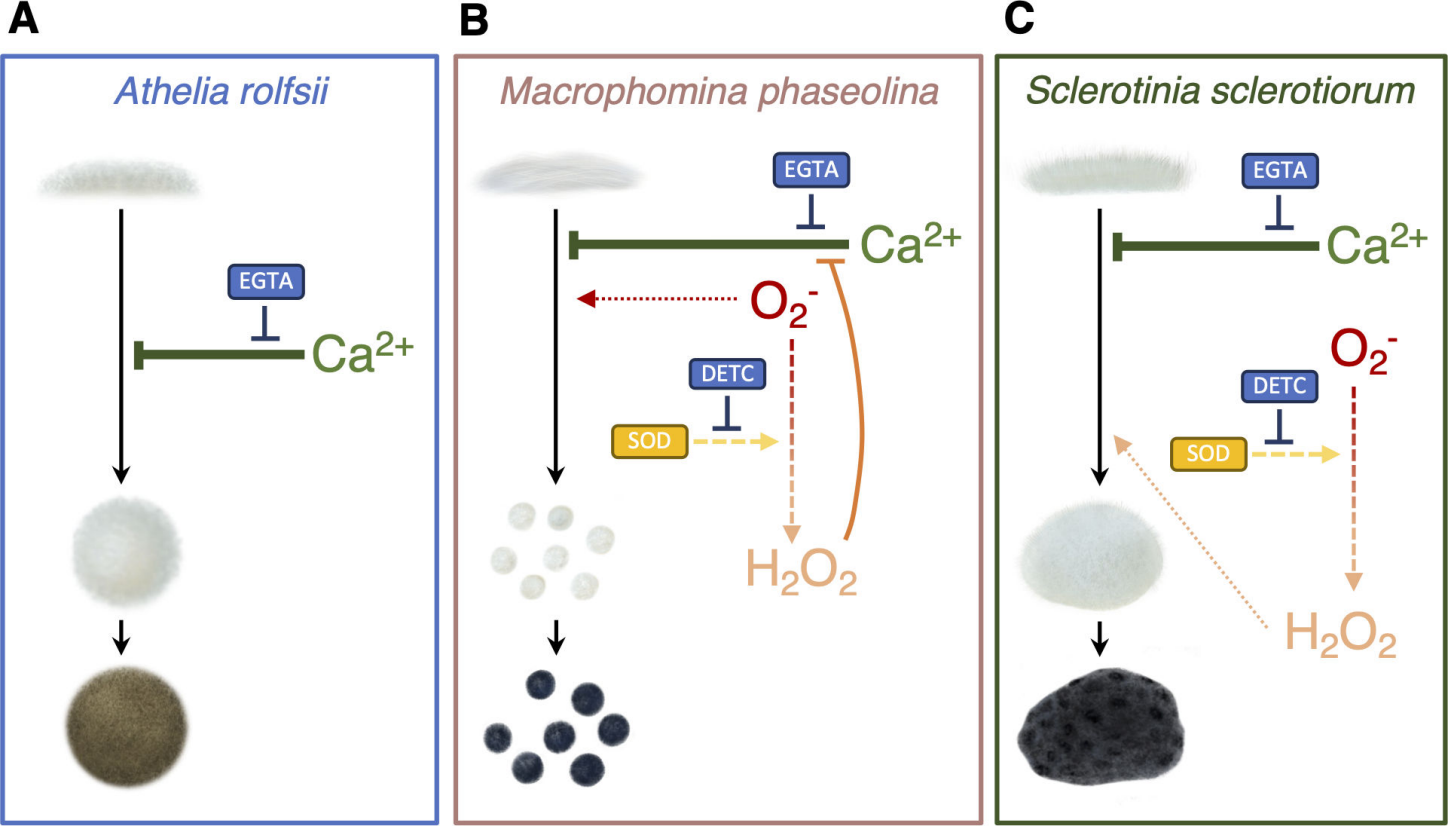
Schematic models of sclerotia formation for *A. rolfsii*, *M. phaseolina*, and *S. sclerotiorum*. (**A**) For *A. rolfsii*, only Ca^2+^ negatively regulates in the hyphal differentiation to sclerotia formation. (**B**) For *M. phaseolina*, Ca^2+^ negatively regulates in the hyphal differentiation to microsclerotia formation. On the other hand, DETC blocks the transition from O_2_^−^ to H_2_O_2_, and the accumulation of O_2_^−^ stimulates more microsclerotia formation. Meanwhile, the addition of H_2_O_2_ partially rescues the suppression effect of Ca^2+^. (**C**) For *S. sclerotiorum*, Ca^2+^ negatively regulates in the hyphal differentiation to sclerotia formation. On the other hand, DETC blocks the generation of H_2_O_2_, leading to less sclerotia formation. Meanwhile, the addition of H_2_O_2_ stimulates more sclerotia formation. The suppression effect of Ca^2+^ appears to be stronger than the stimulation effect of O_2_^−^ and H_2_O_2_ in the cases of *M. phaseolina* and *S. sclerotiorum*, respectively.

However, for *M. phaseolina*, the CaCl_2_–DETC treatment partially restored the suppression effect of CaCl_2_, similar to ETGA but in a weaker attitude (Fig. 6B). Interestingly, the CaCl_2_–H_2_O_2_ treatment also partially rescued the suppression effect of CaCl_2_, similar to CaCl_2_–ETGA, and the restoration was even stronger than CaCl_2_–DETC. The results indicated that both Ca^2+^ and O_2_^−^ may work on the independent signaling hierarchy to influence hyphal differentiation to microsclerotia formation of *M. phaseolina*. Moreover, although H_2_O_2_ was not a stimulus for microsclerotia formation, it may interfere the Ca^2+^ influx, similar to EGTA (Fig. 8).

As for *S. sclerotiorum*, the addition of DETC decreased the sclerotial number likely due to the lack of H_2_O_2_ (Fig. 7B). Furthermore, the CaCl_2_–H_2_O_2_ treatment did not rescue the suppression effect of CaCl_2_. As previous literature and our experiments both supported that exogenous H_2_O_2_ increased the sclerotial number, the results suggested that both Ca^2+^ and H_2_O_2_ may work on the stage from hyphal differentiation to sclerotia formation of *S. sclerotiorum*, but Ca^2+^ appeared to be downstream of H_2_O_2_ or have stronger effect than H_2_O_2_ (Fig. 8). Collectively, the inclusion of *M. phaseolina* and *S. sclerotiorum* supported that the CaCl_2_–ROS interaction assay was technically feasible, and the results collectively support that Ca^2+^, rather than O_2_^−^ or H_2_O_2_, influences the hyphal differentiation to sclerotia formation of *A. rolfsii*.

## DISCUSSION

The generalization of high-throughput sequencing technology has made RNA-Seq a powerful tool for studying the transcriptomics of sclerotia development. There are several fungal systems, such as *M. phaseolina*, *R. solani* AG-1A, and *V. dahliae*, being studied using not only transcriptomics but also culture assays (37, 38, 45). However, being one of the earliest fungi included in the culture assay for sclerotia formation, the transcriptomics of *A. rolfsii* remains unexplored. Therefore, this study was motivated to perform a time-scale RNA-Seq to understand the gene expressions at four developmental stages during sclerotia formation of *A. rolfsii*.

While the ROS-related gene functions were active during the four stages, genes involved in generating ROS (O_2_^−^ and H_2_O_2_) were mostly steady or down-regulated from the mycelial stage (S0) to the initial stage (S1). Moreover, genes involved in scavenging the ROS were mostly steady or up-regulated from the initial stage (S1) to the mature stage (S3). These observations suggested that ROS may not be the key stimulus or signal clue to initiate hyphal differentiation to sclerotia formation for *A. rolfsii*. In the ROS assay with exogenous antioxidants or oxidants, the results further supported that antioxidants (ascorbic acid or DTT), SOD inhibitors (DETC, NaN_3_, or SDS), and H_2_O_2_ cannot affect sclerotia formation. Instead, only GSH and CaCl_2_ reduced sclerotia formation of *A. rolfsii*, likely due to the facilitation of Ca^2+^ influx into hyphae. In the parallel comparison systems of *M. phaseolina* and *S. sclerotiorum*, the results supported that O_2_^−^ for *M. phaseolina*, H_2_O_2_ for *S. sclerotiorum*, and the CaCl_2_–ROS interaction for *M. phaseolina* and *S. sclerotiorum* can affect sclerotial number. However, Ca^2+^ appeared to be the only factor tested in this study that can affect the sclerotia number of *A. rolfsii*.

The model of ROS-dependent sclerotia formation was initiated based on the observation of more lipid peroxidation in sclerotia than mycelia of *A. rolfsii* (34). Later, Sideri and Georgiou (16) reported that light- and Fe-dependent oxidative stress, particularly the increase of H_2_O_2_, was associated with the increase of sclerotia. However, in the study by Sideri and Georgiou (16), *A. rolfsii* remained capable of forming sclerotia even in the absence of light or Fe; moreover, the addition of H_2_O_2_ did not increase the sclerotial number until the H_2_O_2_ concentration was raised above 4.5 mM. The report also showed that undifferentiated and young mycelia actually had the highest H_2_O_2_ amount, and it reached the lowest concentration at the transit into the initial stage of sclerotia formation regardless of light or Fe condition (16). The observation of H_2_O_2_ dropped down toward the mature stage (16) may be explained by our transcriptomic results, which showed the gene expressions of SOD and CAT increased from the S1 to S3 stage, likely to detoxify the ROS stress. In other words, H_2_O_2_ may not be the key signal clue to initiate or influence sclerotia formation of *A. rolfsii*. In a subsequent study, Papapostolou and Georgiou compared the H_2_O_2_ amount between sclerotioenic and non-sclerotiogenic isolates of *A. rolfsii*. The results showed that sclerotioenic isolates exhibited higher H_2_O_2_ than non-sclerotiogenic isolates both intracellularly and extracellularly. However, H_2_O_2_ amount at the four stages of sclerotia formation was measured without statistical analysis to support if the sclerotia initial stage was higher than the undifferentiated mycelia stage (66). Therefore, whether the difference in the H_2_O_2_ amount between the sclerotioenic and non-sclerotiogenic isolates was the cause or a confounding factor requires more experiments to uncover.

While another study showed that exogenous 1- to 2-mM H_2_O_2_ reduced sclerotial number of *A. rolfsii* and over 4-mM H_2_O_2_ increased sclerotial number (36), our experiments showed that H_2_O_2_ above 2 mM suppressed the mycelial growth. Because it has been pointed out that mycelial growth and sclerotia formation were negatively correlated (67), experimental conditions that alter the mycelial growth would make it hard to state the direct effect of H_2_O_2_ on sclerotia formation. In this study, the H_2_O_2_ effect on sclerotia formation was evaluated both by preparing H_2_O_2_-amended media and by immersing mycelial with H_2_O_2_ solution at 1.5 mM, which is a concentration not affecting mycelial growth, and there was no difference regarding sclerotia number. Collectively, although most studies have pointed out the association between H_2_O_2_ and sclerotia formation of *A. rolfsii*, it may not be conclusive to state the role of H_2_O_2_ as a signal clue for the hyphal differentiation to sclerotia formation of *A. rolfsii*.

Among several antioxidants and oxidants tested in this study, only GSH reduced the sclerotial number of *A. rolfsii*. In the following tests with CaCl_2_ and the Ca^2+^–chelator EGTA, the results supported the importance of Ca^2+^ in reducing sclerotia, and microsclerotia formation remained conserved for *A. rolfsii*, *M. phaseolina*, and *S. sclerotiorum*. However, the suppressing effect of Ca^2+^ can be interfered by O_2_^−^ and H_2_O_2_ in *M. phaseolina* and *S. sclerotiorum*, respectively, but not in *A. rolfsii*. Therefore, *A. rolfsii* may be a unique fungal system for the future study of the mechanism of Ca^2+^ decreasing the number of sclerotia formation, while the other two may be useful to understand the interacting effects of Ca^2+^and ROS on the number of sclerotia and microsclerotia formation.

## Data Availability

The RNA-Seq data were deposited in the NCBI BioProject PRJNA1042918. (According to the NCBI depository option, the data will be publicly available upon the release date or the manuscript acceptance date, whichever comes the first.)
